# Centering the Organizing Center in the *Arabidopsis thaliana* Shoot Apical Meristem by a Combination of Cytokinin Signaling and Self-Organization

**DOI:** 10.1371/journal.pone.0147830

**Published:** 2016-02-12

**Authors:** Milad Adibi, Saiko Yoshida, Dolf Weijers, Christian Fleck

**Affiliations:** 1 Laboratory of Systems and Synthetic Biology, Wageningen University, Wageningen, the Netherlands; 2 Laboratory of Biochemistry, Wageningen University, Wageningen, the Netherlands; Lund University, SWEDEN

## Abstract

Plants have the ability to continously generate new organs by maintaining populations of stem cells throught their lives. The shoot apical meristem (SAM) provides a stable environment for the maintenance of stem cells. All cells inside the SAM divide, yet boundaries and patterns are maintained. Experimental evidence indicates that patterning is independent of cell lineage, thus a dynamic self-regulatory mechanism is required. A pivotal role in the organization of the SAM is played by the *WUSCHEL* gene (WUS). An important question in this regard is that how WUS expression is positioned in the SAM via a cell-lineage independent signaling mechanism. In this study we demonstrate via mathematical modeling that a combination of an inhibitor of the Cytokinin (CK) receptor, *Arabidopsis* histidine kinase 4 (AHK4) and two morphogens originating from the top cell layer, can plausibly account for the cell lineage-independent centering of *WUS* expression within SAM. Furthermore, our laser ablation and microsurgical experiments support the hypothesis that patterning in SAM occurs at the level of CK reception and signaling. The model suggests that the interplay between CK signaling, WUS/CLV feedback loop and boundary signals can account for positioning of the *WUS* expression, and provides directions for further experimental investigation.

## Introduction

All the aerial plant parts are generated by the shoot apical meristem (SAM) situated at the plant apex. The SAM is formed during embryogenesis and in dicotyledonous angiosperms, such as the model plant *Arabidopsis thaliana*, it contains three layers of stem cells in the three outermost cell layers [1, 2]. Clonal studies indicate that each layer contains about three long lived stem cells [3] in the central zone (CZ), which is marked by a lower cell division rate. Daughter cells of the stem cells that stay in the CZ replenish the stem cell pool, whereas daughter cells that are placed towards the peripheral zone (PZ), which is marked by a higher cell division rate, enter differentiation and form organ primordia. The shape and the domain structure of the SAM are kept unchanged, although all cells continuously divide and differentiating stem cell daughters leave the meristem. Cell tracking and ablation experiments demonstrate that the fate of each cell is determined by its current position and not by lineage specific heritage, highlighting the importance of cell-cell communication [4]. Due to its changing cellular context, pattern formation of the shoot meristem does not rely on a stable point of reference, but rather occurs in a self organized manner [1].

**Fig 1:**
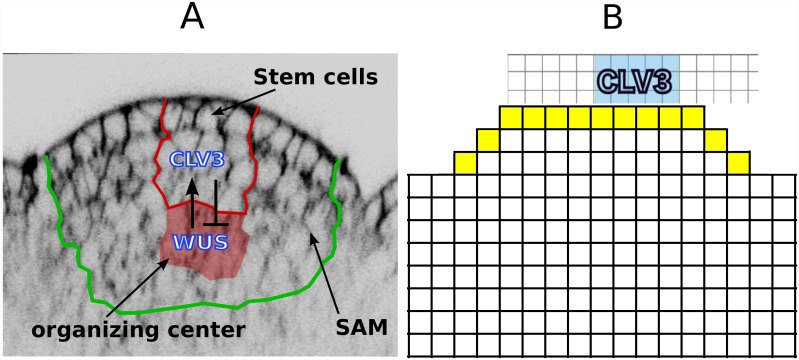
SAM architecture and its representation in the model. (A) An image of the SAM and the immediate surrounding area. The regions of interest are marked with colored boundaries. (B) Schematic representation of WUS and CLV domains; the three dimensional SAM, consisting of cells of various shapes and sizes, is modeled by a two dimensional grid consisting of identical blocks representing cells. The field of cells extend farther in lateral and basal directions. S2 Fig depicts the complete cellular template used for simulations.

Genetic studies mainly in *Arabidopsis* reveal that the WUSCHEL (WUS) and CLAVATA3 (CLV3) feedback loop is a pivotal regulator of stem cell number [1, 2, 5, 6]. A small cell group underneath the stem cells named organizing center (OC) expresses the transcription factor WUS that maintains the stem cell in two ways. First, WUS protein moves into the stem cells, presumably through intercellular plasmatic bridges, called plasmodesmata [7]. In the stem cells, WUS directly binds to the promoter of *CLV3* and promotes transcription, in addition to maintaining pluripotency through a yet unidentified mechanism [1]. *CLV3* encode a small extracellular signal peptide that binds to receptor kinase complexes, including CLV1, and triggers an intracellular signal cascade that downregulates *WUS* transcription [1, 8]. This negative feedback loop between OC and stem cells provides a mechanistic framework to keep the number of stem cells constant [1], see Fig 1A. Second, in the OC cells, WUS directly represses transcription of *ARABIDOPSIS RESPONSE REGULATOR 7* (*ARR7*) and 15 (*ARR15*) genes [9], which encodes intracellular inhibitors of response to the plant hormone Cytokinin (CK), thereby promoting cellular CK response [10]. Hence, the question of how WUS expressions is centered and restricted within the SAM, becomes a key question in studying the stem cell homeostasis in the SAM. Several lines of evidence further indicate that CK is an important factor in shoot meristem regulation: first, mutants deficient in CK biosynthesis, reception, or overexpressing CK degrading enzymes, have a reduced SAM size [2, 11, 12]. Second, the CK receptor, Arabidopsis histidine kinase 4 (AHK4), is expressed in the meristem center, overlapping with the OC in its distal part [13]. The receptor is involved in the upregulation of WUS expression via exogenously supplied CK at relatively high levels, and it has been assumed to confer WUS regulation also at endogenous CK levels. CK response, measured by the reporter pTCS, peaks at the OC [14] in agreement with WUS enhancing CK response in these cells. Based on the expression pattern of the transcription factor SHOOTMERISTEMLESS (STM) that promotes expression of the CK synthesis gene *ISOPENTENYL TRANSFERASE* 7 (*IPT7*), CK is probably produced broadly throughout the shoot meristem, although direct evidence is still missing [15]. Furthermore, immunodetection of CKs suggest a rather broad and uniform distribution throughout the shoot meristem in *Sinapsis alba*[16]. Recent findings in rice indicate that activation of CK (clipping of a ribose residue) by the LONELY GUY (LOG) enzyme is confined to the 2-3 outermost cell layers of the shoot meristem, and it has been discussed whether active CK is locally produced in the shoot meristem and moves from the top downwards [2, 17]. In *Arabidopsis*, there are eight LOG homologs. One of them, LOG4 is specifically expressed in the L1 layer, but the expression patterns of the other *LOG* genes are unknown and at least some of the other LOG genes seem to be also expressed in the shoot meristem [13, 18, 19].

### Previous modeling approaches

The presence of cell lineage-independent self-organization suggests that the internal structure is maintained by a network of signals that interact with each other and can create stable isolated peaks of concentration. One theoretical approach that was successfully applied to explain self-regulated pattern formation in developmental biology is the reaction-diffusion scheme first introduced by Turing in 1952 [20], and has since been applied to various fields of developmental biology [21–23]. Most of the applications of the reaction-diffusion scheme in pattern formation in biology have been in the form of activator-inhibitor systems. In its simplest form an activator-inhibitor system consists of two interacting diffusing molecule [24]. Modeling the self-organized pattern formation in the SAM has been subjected to various modeling approaches among which, activator-inhibitor models have been the main approach. Jönsson *et al.*[25] were the first to model the stem cell regulation in the SAM using an activator-inhibitor model. This pioneering work demonstrated that an activator-inhibitor based system can account for the observed expression of WUS in the SAM. Hohm *et al.* (2010), developed the first model that incorporates complete feedback between CLV3 and WUS. This model not only reproduced the expression patterns of WUS and CLV3 observed in the wildtype SAM but also some mutants and gene up and down-regulation phenotypes, further demonstrating the capability of activator-inhibitor models in accounting for SAM organization [26]. In [27] Fijuta *et al* implement an activator-inhibitor-based model of WUS/CLV3 regulation in a growing and dividing cellular template. Their work presents a model that is stable against perturbations caused by cellular growth and division., albeit lack of data has led to various assumptions, The activator-inhibitor based models can account for some fundamental aspects of stem cell regulation within the SAM. These models, like other spatial models of cellular development, have restrictions regarding the level of detail and the scope of the model. Often it is unavoidable to consider the input of other processes as pre-patterns or hypothetical components. Despite these limitation these models have been successful in providing an integrated view of the available experimental data regarding SAM. patterning. The hypothetical components of these models point out gaps in our biological knowledge that need to be addressed in order to obtain a mechanistic understanding of the SAM stem cell regulatory mechanism.

The aforementioned models focus on how the WUS expression pattern can emerge from the interaction of network components within the SAM. The computational models of SAM organization however, have not been limited to self-organizing systems, other models have focused on investigating the interplay between gene expression patterns rather than self-organized pattern formation [13, 14, 28]. These models focus on the experimentally demonstrated interactions between the WUS/CLV3 patterns and CK signaling/perception network [13, 14, 28]. For instance, Yadav *et al.*[28] investigate a model that relates CK perception via AHK4 receptor to pattern formation in the SAM. In this model CK is induced by an AHK4/CK signal, which is produced at the center of OC. The expression zones (i.e. binary expression templates) of WUS, CLV3, and KAN1 are restricted to CZ, OC, and PZ, respectively. Given these inputs the model can robustly establish the spatial patterns of WUS, CLV3 and KANDI1. Furthermore, these patterns can withstand perturbations caused by cell growth and division. In the aforementioned work the localized expression of AHK4 at the center of the OC is fundamental for correct patterning of WUS. This group of experimental and computational works, consistently propose that the patterning of OC takes place at the level of CK reception and signaling. Consequently, this implies that the self-organizing properties of the OC, can arise from the underlying CK signaling/perception pattern.

### Aim of this study

The capability of activator-inhibitor networks in accounting for SAM patterning has been already demonstrated. In general, the experimental identification of network components has been a major challenge in application of reaction-diffusion models in biology. Particularly in the plant field, it remains a major challenge to demonstrate the existence of reaction diffusion networks experimentally.

In our context this highlights the importance of motivating the pre-patterns of a model by known biological knowledge as much as possible; when pre-patterns are abstract and cannot be directly linked to the known biological mechanisms, the task of experimental identification of network components is complicated. In contrast, when these assumptions are motivated by experimental observations, they can be more readily investigated via experimentation. As discussed earlier, theoretical and experimental data point towards CK signaling and perception as a fundamental factor in patterning of the SAM [13, 14, 28]. Here we aim to expand upon the current state of research and avoid incorporation of abstract assumptions in our model, by utilizing the available data as much as possible. Our model links WUS/CLV3 feedback loop to an activator-inhibitor system based on CK signaling. We demonstrate that these components function together to position WUS expression at the OC.

## Results

### The Model

In order to investigate the apical-basal position and the lateral extension of the OC within the shoot meristem, we chose a two dimensional model of a longitudinal section. In our model, mobile signals are free to diffuse out of the SAM and into the surrounding cells (Fig 1). The system is described by a set of coupled non-linear ordinary differential equations on a discrete grid, where the grid points represent the individual cells. Hence, cells are assumed to be spatially uniform and intracellular concentration gradients are not taken into account, which is considered a reasonable simplification due to the difference in timescale between cytosolic mobility (fast) and the actual pattern formation process, i.e., gene expression (slow). Therefore, we use the term diffusion not in its actual physical but rather in an effective sense, meaning unbiased bi-directional spread of molecules between cells through special openings termed plasmodesmata or via the apoplast. Moreover, for simplicity, we assume that the dynamics of the WUS/CLV3 regulatory system arising from the assumed reaction-diffusion system are sufficiently faster than the cell division rate in the SAM. Therefore, the essential features of the patterning process of the aforementioned system can be well described by a static model that does not incorporate cell division or growth.

### Facts and assumptions underlying the model

The proposed model is based on the following published results:

- CK binds to the AHK4 receptor, which in turn causes phosphorylation of both type-A and type-B ARRs via arabidopsis histedine phosphotranfer proteins (AHPs) [29]. In absence of CK the receptor functions as a phosphatase. [30, 31].
- Type-B ARRs are transcription factors that activate transcription of CK response genes, including type-A ARRs [32].
- Type-A inhibit type-B ARR function, the precise mechanisms has yet to be determined [10, 33]. In general two hypotheses exist regarding the inhibition of type-B ARRs via type-A ARRs. Evidence suggests that type-A ARRs inhibit type-B ARRs via repression of upstream CK signaling. In addtition it has been proposed that type-B repress type-A via competition for phosphate molecules [34].
- There is feedback loop between *WUS* and *CLV3* genes, where WUS moves from the OC into the stem cells and activates the transcription of the *CVL3* gene. The CLV3 peptide is mobile and inhibits the expression of WUS [1, 35].
- Expression of WUS is activated by CK signaling [13], presumably via canonical type-B ARRs effector genes. Additionally WUS represses the expression of type-A ARRs [9], thus promoting CK signaling.

In addition, our model incorporates the following assumptions:

- The SAM consists of equivalent cells that have the potential to express all genes included in the model. The exception is the epidermis (L1 layer), which is assumed to be different from the rest of the cells in the SAM. This means that in our model the identity of the L1 cell layer is not determined via the proposed self-organizing mechanism.
- We hypothesize a molecule (L1 signal) that is supplied by the uppermost cell layer (L1) and diffuses downwardly establishing a gradient. The presence of this molecule is necessary for the cells to be able to respond to WUS signal by producing CLV3. Such a molecule has been identified by Knauer *et al* (2013), who charactrized a microRNA, miR394, that is produced at the L1 layer and is required for establishment of CLV3 expression. In our model the L1 signal is necessary for cells to be able to respond to WUS and establish stem cell identity [36].
- A diffusing inhibitor and a self-activating component are essential parts of pattern forming activator-inhibitor mechanisms [24]. Currently there is no evidence of such an inhibitor involved in SAM patterning; our trials show that several molecules within the model can be assumed to act as an inhibitor or to induce an inhibitor. For example, type-A ARRs appear as a plausible candidate for the inhibitor within the model. It is known that type-A ARRs inhibit CK signaling [10]. In the model the inhibitor is assumed to be downstream of the type-A ARRs. This is because the to fulfill the role of the inhibitor within an activator-inhibitor system, type-A ARRs have to be highly mobile signals. In absence of evidence regarding the mobility of these molecules, we assumed that the inhibitory function is conveyed via a highly mobile intermediate, factor X. Thus in our model two mechanisms exist for inhibition of type-B by type-A ARRs, via phosphate competition and via factor X.
- We assume type-B ARRs promote CK signaling via direct induction of AHK4. Experimental results presented on Arabidopsis eFP browser [37], (data from AtGenExpress project [38]) show that application of Zeatin leads to significant up-regulation of AHK4 levels. For the model, this assumed interaction constitutes the autocatalytic loop of the activator-inhibitor subnetwork.
- In our reductionist approach, we do not distinguish between mRNA and protein of the genes unless it is essential in addressing the question at hand. Considering the expression pattern of *CLV3* mRNA [1] and its demonstrated inhibitory effect at the OC, it becomes apparent that CLV3 elicits a signal that travels further than it its mRNA. This is reflected in the model by distinguishing CLV3 mRNA and protein. The mRNA is assumed to be immobile while the protein is able to diffuse.
- In the model we assume that phophotransfer from the receptor to the ARRs are sufficiently fast processes compared to gene expression. Therefore the phosphotransfer is implemented using a quasi-steady-state assumption. See detail in Material and methods.

Reaction-diffusion modeling of the SAM has a history of more than a decade and the model presented in this work is inspired and motivated by earlier modeling efforts. It utilizes concepts and components (experimentally verified as well as hypothetical) put forward in earlier works In particular, factor X is a universal component of activator-inhibitor models of the SAM [25–27, 39]. As the inhibitor in an activator-inhibitor systems, models consistently predict it to be a fast diffusing molecule with an expression pattern centered around SAM. To date evidence for a molecule that fulfills the role of such inhibitor and matches its predicted expression pattern has not emerged. Similarly the concept of L1 signal was first established by Joensson *et al* in [25]. In later works this was utilized as a signal defining the lateral expression of WUS [40], as well as in an apical-basal setting, as a cofactor that along with WUS is required for production of CLV3, in both two-dimensional [7], and three-dimensional [28]settings. In our model the L1 signal is essentially the same as the in latter; an apical basal signal required for CLV3 inductio in response to WUS. As already pointed out in [28], the strongest evidence for the existence of such a signal comes from the observation that in *pCLV3::WUS* both *WUS* and*CLV3* are expressed in the uppermost three cell layers of the SAM [41].

### Model equations

Integrating the above stated experimental observations and additional assumptions in a mathematical model, we arrive at the following system of non-dimensionalized coupled ordinary differential equations:

$$\begin{array}{ccc} \frac{dB_{ij}}{dt}\; & = & \;\frac{k_{1}\Gamma_{ij}B_{ij}}{\left(1+k_{6}X_{ij}\right)}-k_{7}B_{ij} \end{array}$$ (1)

$$\begin{array}{ccc} \frac{dA_{ij}}{dt}\; & = & \;\frac{k_{8}\Gamma_{ij}B_{ij}}{\left(1+k_{6}X_{ij}\right)\left(1\;+\;k_{9}W_{ij}\right)}-k_{10}A_{ij} \end{array}$$ (2)

$$\begin{array}{ccc} \frac{dR_{ij}}{dt}\; & = & \;\frac{k_{11}\Gamma_{ij}B_{ij}}{1+k_{6}X_{ij}+k_{12}\Gamma_{ij}B_{ij}}-k_{13}R_{ij} \end{array}$$ (3)

$$\begin{array}{ccc} \frac{dX_{ij}}{dt}\; & = & \;k_{14}\Gamma_{ij}A_{ij}-k_{15}X_{ij}+d_{1}\hat{D}X_{ij} \end{array}$$ (4)

$$\begin{array}{ccc} \frac{dW_{ij}}{dt}\; & = & \;\frac{k_{16}\Gamma_{ij}B_{ij}}{\left(1+k_{6}X_{ij}\right)\left(1+k_{17}{\text{Cs}}_{ij}\right)}-W_{ij}+d_{2}\hat{D}W_{ij} \end{array}$$ (5)

$$\begin{array}{ccc} \frac{dC_{ij}}{dt}\; & = & \;\frac{k_{18}L_{ij}W_{ij}}{1+k_{19}W_{ij}}-k_{20}C_{ij} \end{array}$$ (6)

$$\begin{array}{ccc} \frac{d{\text{Cs}}_{ij}}{dt}\; & = & \;k_{21}C_{ij}-k_{22}{\text{Cs}}_{ij}+d_{3}\hat{D}{\text{Cs}}_{ij} \end{array}$$ (7)

where we defined:

$$\begin{array}{ccc} A:=[\text{type}-A\;\text{ARR}]; & & \;B:=[\text{type}-B\;\text{ARR}]\\ C:=[\text{CLV}3\;\text{mRNA}]; & & \;\text{Ck}:=[\text{Ck}]\\ \text{Cs}:=[\text{CLV}3\;\text{peptide}]; & & \;R:=[\text{AHK}4]\\ W:=[\text{WUS}]; & & \;X:=[\text{Inhibitor}]\\ \;L:=[L1\;\text{signal}] & & \end{array}$$

and

$$\begin{array}{c} \Gamma_{ij}:=\frac{{\text{Ck}}_{ij}R_{ij}}{\left(1+k_{2}{\text{Ck}}_{ij}+k_{3}R_{ij}\right)\left(1+k_{4}A_{ij}+k_{5}B_{ij}\right)}. \end{array}$$ (8)

The subscript *ij* denotes the position *x* = *x*_*ij*_ on the grid, where i and j refer to row and column indices respectively. *L*_*ij*_ and *Ck*_*ij*_ refer to the L1 signal and the CK expression profiles derived in material and methods. The function Γ is the transfer function for the two step phosphorelay from CK binding to the phosporylation of the ARRs. It comprises the kinase and phosphatase activity of the AHK4 receptor, the phosphorelay via the AHPs and the competition between the type-A and type-B ARRs for the AHPs, for the derivation see material and methods. The spatial coupling between the grid points is achieved by the diffusion operator $\hat{D}$ (see material and methods). We use reflecting boundary conditions for the apical side. The basal and lateral boundary is not well defined; we use boundary conditions which are, for simulation purposes, equivalent with using an infinite domain for the apical-basal dimension; in the numerical simulations the grid is extended in basal and lateral directions until the concentrations decay to almost zero; this makes the boundary condition at the basal and lateral end of the grid irrelevant and provides a good approximation for the *in vivo* SAM. We close the domain basally and laterally using reflecting boundary conditions. All simulations, unless stated otherwise, were carried out in a cell grid where the meristemic dome is represented by three cell layers that are 9, 11 and 13 cells wide. The uppermost cell layer and the laterally outermost cells of the other two layers make up the L1 layer (Fig 1B). We always checked that the grid is large enough to approximate an infinite domain in the described manner. In the following figures, the area of the grid that contains no information has been cropped.

#### Mobility of molecules in the model

In a model, the ‘assigned’ mobility of molecules can occur at the level of any intermediate components that are not explicitly present in the model. Moreover, in such a case, often, mRNA and protein of a gene are considered a single identity, hence in reality, the assigned mobility can occur at the level of either mRNA or the gene.

The correct patterning of the model depends on mobility of WUS, CLV3 peptide, L1 signal and factor X. in the model WUS needs to me mobile to reach L1 layer and trigger the expression of *CLV3*. The mobility of WUS protein has been demonstrated previously and WUS protein is detected at L1 layer [35]. In order to inhibit WUS at the OC, CLV3 is required to be mobile in the model. Similarly the intercellular movement of CLV3 peptide has been established [8]. As an inhibitor in an activator-inhibitor system, the mobility of factor X is required for model functionality. As mentioned earlier, a feasible candidate for the role of L1 signal is miRNA394, which has been shown to act as a mobile signal. [36]

### Model analysis

We tested whether the proposed model can account for the observed patterns of CLV3 and WUS in the SAM and whether it can reproduce known experimental results, which are relevant to the patterning process. To this end, we performed numerical experiments: we examined whether the model is capable of reproducing the observed phenotypes wildtype SAM as well as various non-wildtype scenarios including mutants and overexpression lines. Because we use a mechanistic model, we can map experimental manipulations directly to the parameters of the model. Therefore the non-wildtype scenarios can be implemented by changing the model component that corresponds to the specific mutation, overexpression, etc. For instance, a knock out mutation is implemented by setting the production rate of the affected gene to zero. To simulate the ablation scenarios, the appropriate changes are applied to the wildtype system at the steady sate. Once the system reaches a steady state again, the resulting expression patterns are compared against the experimental observations.

#### Model subnetwork structure

**Fig 2:**
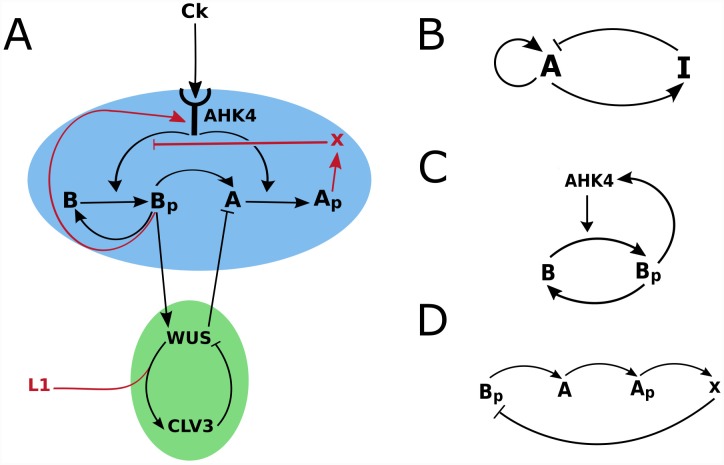
Two coupled sub-networks and boundary information define WUS and CLV3 expression domains in the SAM. *A* and *B* stand for type-b and type-A ARRs. *A*_*p*_ and *B*_*p*_ denote phosphorylated type-B and type-B ARRs. (A) The model can be divided into the CK signaling (blue) and WUS/CLV3 (green) sub-networks combined with boundary morphogens (L1 and CK). The former determines the position of the WUS domain via a self-organizing system while the latter specifies the CLV3 domain, taking the WUS domain as an input. The network consists of interactions and molecules that are based on published experimental data (black arrows and letters) and hypothetical interactions and molecules (red arrows and letters). Parts of the CK signaling sub-network correspond to the components of the (B) classical activator/inhibitor system; (C) the network component corresponding to the autocatalytic activator and (D) to the activation/inhibition interactions.

The model in essence consists of two coupled subnetworks: WUS/CLV3 (Fig 2A, green shaded) and the CK signaling (Fig 2A, blue shaded). In addition boundary information is supplied by CK and the L1 signal (Fig 2A, red arrows; also see S1 Fig for the profiles). Parts of the CK signaling network correspond to components of a classical activator/inhibitor system (Fig 2A). The AHK4/B/B_p_ part of the network acts as an autocatalytic activator (Fig 2C), while the pathway leading from B_p_ to X, fulfills the role of induction of the inhibitor by the activator (Fig 2D). The CK gradient confines the domain of pattern formation to the upper part of the meristem. The activator-inhibitor network is coupled to the WUS/CLV3 subnetwork via an incoherent feed-forward loop (B_p_/WUS/A) which specifies WUS expression by type-B ARR. The WUS/CLV3 subnetwork generates the expression domain of CLV3. Boundary information supplied by the L1 signal determines the correct orientation of the CLV3 expression in apical-basal direction. S2 Fig contains examples of the model out put in the uncropped simulation grid.

#### Robust reproduction of the wildtype expression patterns of the genes included in the model

**Fig 3:**
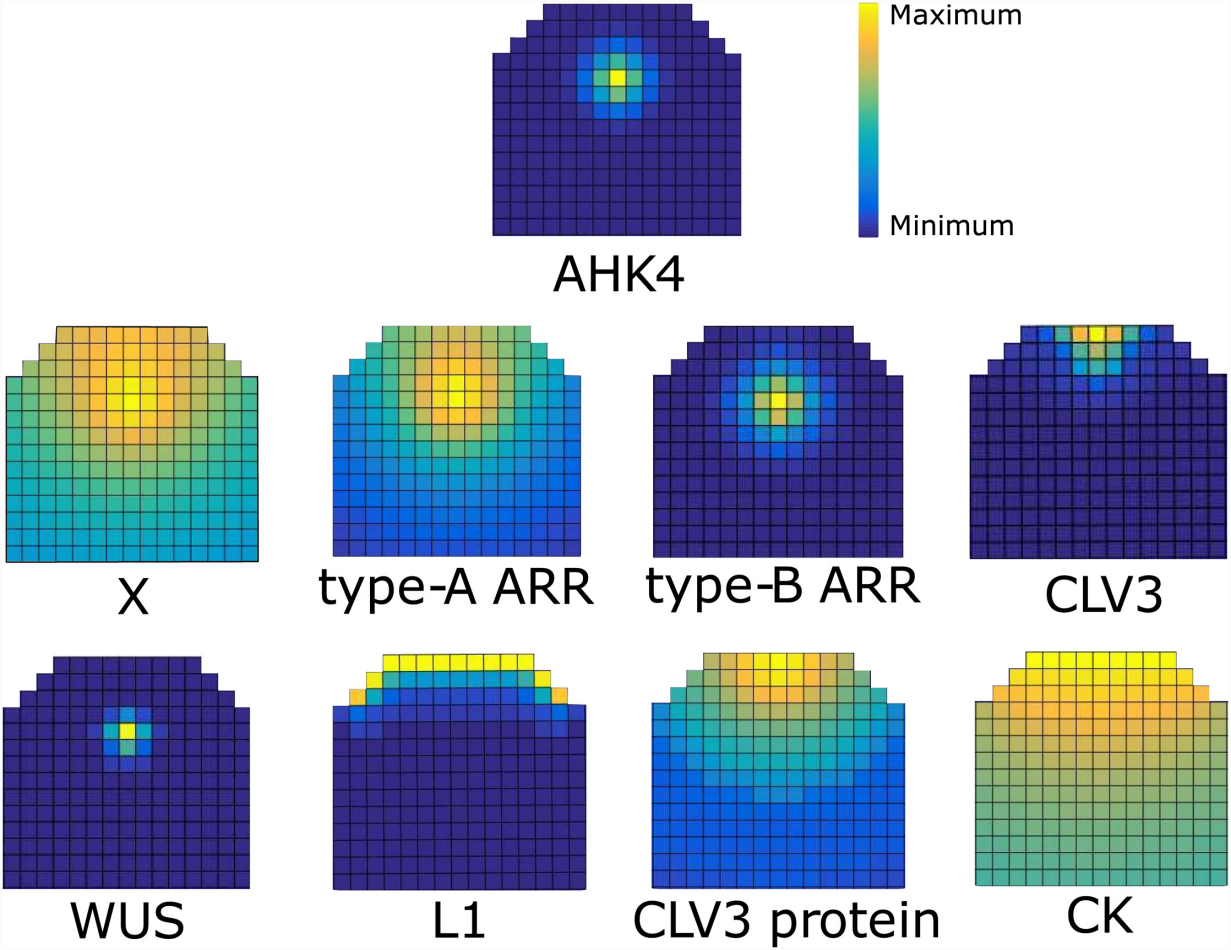
Wildtype expression pattern of the molecules in the model. The relative levels in each figure are depicted by a color spectrum shown by the color bar in the figure. For examples of models out put in an uncropped template see S2 Fig.

The *sine qua non* for the model is of course whether the observed wildtype pattern can be established and maintained. The simulated wildtype pattern is shown in Fig 3. WUS is present in a high concentration in a small region at the center of SAM, which in both lateral and apical basal directions corresponds to the observed experimental pattern [1]. In our model the lateral position of a single WUS peak is always at the center, whereas the apical basal position depends on the region defined by CK. The size of the WUS domain depends on the dynamics of the activator-inhibitor subnetwork as well as inhibition from CLV3.

We investigated the effect of WUS mobility on model output by setting WUS diffusion to zero. This cell-autonomous version of WUS is only detected in the OC and is absent from the upper cell layers of the SAM, S3A Fig. This results in significant reduction of CLV3 levels and misplacement of its domain, S3C Fig in comparison to wildtype, S3B and S3D Fig. This simulated WUS pattern closely resembles the observed transcriptional pattern of *WUS* in the SAM [35, 42]. In contrast, when WUS mobility is considered in the model the resulting expression extends to the L1 layer, S3B and S3D Fig. The predicted patterns of AHK4 and WUS by the model, overlap in the OC. This has been observed experimentally and reproduced by previous models of WUS/CLV3 interactions [14, 43]. Furthermore the model predicts the WUS expression domain to constitute a sub-section of the broader AHK4 domain. This is in agreement with the observed distribution of *WUS::DsRed-N7* in the apical half of the AHK4 receptor domain marked by *AHK4::GFP*, in inflorescence meristem [13]. Patterns of type-A and type-B ARRs, see Fig 3C and 3D, are comparable to the patterns reported by [14]. It should be noted that the pattern of type-A ARR expression in the model refer to the phohporylated portion of these proteins, while the relevant experimental data primarily consists of GUS reporter and transcriptional marker gene expressions [9, 14, 44]. This complicates the comparison of model output in terms of type-A ARR expression against experimental data. Nevertheless the model predicts that WUS expression domain and the domains associated with CK signaling (AHK4, type-A and type-B ARR expression domains), largely overlap. This is in agreement with experimental observations of ARR5 [13] and AHK4 [13, 14]transcriptional reporters in the SAM, as well as with previous models of mutual interactions between CK signaling and WUS in the SAM [13, 14].

*CLV3* (mRNA) expression is limited to the tip of the meristem, with an expression zone that is wider at the apical end and narrows towards basal limit of CLV3 expression, Fig 3E. This is in agreement with the CLV3 (mRNA) patterns observed experimentally.

We investigated the effects of our choice of representation of meristem and L1 layer on model output. We observed that the model is not dependent on this particular representation; model simulations using several other representations of meristemic geometry and L1 layer, produce the correct output, see S4 Fig.

#### Sensitivity Analysis

In order to analyze the model performance we carried out a parameter survey in which we compared the simulated patterns against experimental observations of WUS and CLV3 pattern in the SAM (see Material and Methods, S5 Fig). For the subset of tested parameters for which the model performed in sufficient agreement with the experimental data we analyzed their sensitivity (see Material and Methods).

The model displays little sensitivity to variations of most of the parameters, demonstrating robustness within the defined parameter sub-space, S6 Fig. The model shows high sensitivity to parameters *k*_1_, *k*_6_, *k*_14_ and *d*_2_, which correspond to production rate of type-B ARRs (*k*_1_), phosphorelay inhibition by X (*k*_6_), production of the inhibitor X (*k*_14_) and the diffusion rate of WUS (*d*_2_). The first three parameters (*k*_1_,*k*_6_,*k*_14_) are essential for the correct functioning of the reaction-diffusion system and correspond to the activity of the autocatalytic loop (*k*_1_), inhibitory effect of the inhibitor (*k*_6_) and the production rate of the inhibitor (*k*_14_). The model shows the highest sensitivity to the diffusion rate of WUS *d*_2_ which represents the ratio of WUS diffusion to type-B ARR diffusion. The direct effect of *d*_2_ is to influence the width of the WUS expression peak. Additionally WUS diffusion along with L1 signal determines the expression of CLV3. This double effect of the WUS diffusion rate *d*_2_ on both WUS and CLV3 expression domains explains why it is the most sensitive parameter. Other diffusion/degradation rates in the reaction-diffusion subnetwork do not directly affect the expression pattern of CLV3.

#### The L1 signal has to be confined to a few cell layers

**Fig 4:**
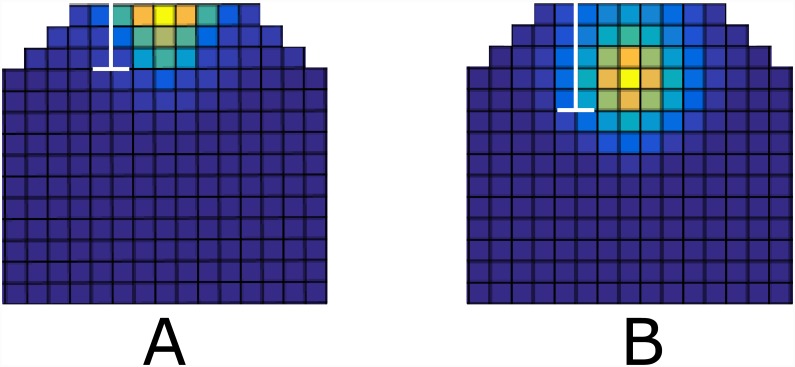
Extension of L1 signal beyond the first three cell layers. The white bar in the figures show the distance at which the concentration of the L1 signal drops to 10% of its initial concentration (A) CLV3 expression in wt. (B) CLV3 expression resulting from the extended L1 signal. the CLV3 mRNA expression extends to organizing center. This has never been observed experimentally in the wildtype SAM, hence the model predict that L1 signal is confined to the upper three cell layers. For examples of models out put in an uncropped template see S2 Fig.

By altering the hypothetical L1 signal we can identify some properties of this signal, which are essential for the correct behavior of the model. By this, we can assess the model hypothesis regarding this directional signal in the SAM, when a candidate for such a signal is identified. For simulations of the wildtype shown in Fig 3, the L1 signal extends to only a few cell layers below the L1 layer as shown in Fig 3G. We examined the scenario where the L1 signals extend farther down the meristem. This can be achieved either by increasing the diffusion rate of the signal and/or by decreasing its degradation rate. The extension of L1 signal results in enlargement of CLV3 domain and presence of CLV3 in the OC (compare Fig 4A and 4B), which is never reported in wildtype. Therefore, the model predicts that in wildtype conditions the directional signal originating in the L1 layer, is confined to the 3-4 uppermost cell layers, which is in good agreement with the spread detected for miR394 [36].

#### Limitation of the CK response profile

**Fig 5:**
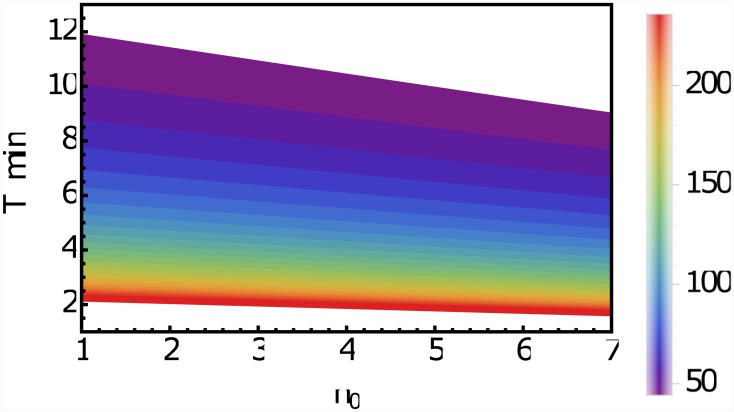
CK lifetime as a function of *D*_*eff*_ and *n*_0_. The average lifetime *τ* of CK in minutes in the meristem and the extension *n*_0_ in cell layers of the CK synthesis zone consistent with the observation of a CK profile covering the upper 25 cell layers of the meristem. The colorbar shows the chosen value of the effective diffusion constant of CK in the meristemic tissue, ranging from 42*μm*^2^ *s*^−1^ to 241*μm*^2^ *s*^−1^ (see text).

For the proposed model it is only important that CK is limited to the upper 20-30 cell layers of the meristem, the actual process by which this is achieved is not important. As there is no experimental evidence that a diffusion or transport barrier—such as a Casperian strip—in this region exists, we analyze the consequences of the assumption that the CK profile is *not* limited by a physical barrier. Because no evidence for a directed transport of CK in the SAM exists, we consider the mobility of CK as a non-directional diffusion-like process. In this case the CK profile is governed by three parameters: the size of the synthesis zone *n*_0_, the average lifetime *τ* of a CK molecule and the effective diffusion rate *D*_*eff*_. Unfortunately, for none of these parameters estimates are available. CK profile was experimentally measured to cover the first 25 cell layers of the meristem [16], i.e., the synthesis regime does not extend beyond this. It seems reasonable to limit it further to the actual meristem [3]. From this follows that $1\leq n_{0}\underset{\approx }{<}7$. The long-distance translocation of CKs is mediated by the xylem and the phloem and has been experimentally investigated [45]. However, for this study the local short-distance mobility of CK across the plasma membrane and the cell wall is important, for which the mechanisms are not well understood [46]. The purine permease family and the equilibrative nucleoside transporter family have been proposed as candidates for CK transporters. While the first can transport free-base CKs in a proton-coupled manner the latter facilitate diffusion of nucleosides along a concentration gradient [47]. In any case, the mobility of CK in the SAM is determined by its diffusion in the cytoplasm and the transport/diffusion across the cell boundaries, where the latter is likely to be the limiting process. In order to obtain an estimate for the upper limit for the effective diffusion rate in the SAM tissue we consider the diffusion of a molecule in the cytoplasm. Based on measurements in E. coli, we find as a rough estimate of the diffusion constant in the cytoplasm $D_{\text{eff}}^{>}\approx 241\mu m^{2}s^{-1}$[48, 49]. An estimate for the lower limit can be obtained by considering the diffusion of molecules within the cell wall [47]. We obtain for CK as lower limit $D_{\text{eff}}^{<}\approx 42\mu m^{2}s^{-1}$. The degradation of CK is catalyzed by CK oxidase/dehydrogenase [3]. It appears that degradation is a highly regulated process, which makes it difficult to say something about the rate. To date the degradation rate of CK in the SAM has not been measured. Therefore, no further information is available. However, we can use these considerations to obtain an idea about the average lifetime of a CK molecule. For a given diffusion rate *D*_*eff*_ and a range *n*_0_ of the synthesis zone the average degradation rate or life-time *τ* of CK follows from limiting the profile to the upper 25 cell layers (see material and methods). The resulting *τ* as a function of *D*_*eff*_ and *n*_0_ is shown in Fig 5. Due to the rough estimates available for the other parameters, there is of course a range for *τ*, but interestingly the deliberations above, point towards a lifetime of CK of the order of a few minutes.

An interesting question in this context is, how the phloem might alter the CK profile. The phloem starts several cell layers below the meristem [50, 51]. A simple physical picture for the CK transport inside the phloem is that of mass transport due to the bulk motion of a fluid (advection). The results of the analysis described in the material and methods suggests that advection via phloem, at a distance of roughly 220*μ*m, hardly affects the CK profile in the meristemic zone, which is rather defined by the diffusion length scale. However, in young plants the situation is quite different. Unless the diffusion length is modified during the growth process, the meristemic zone defined by the CK profile would be unrealistically large. The close proximity of the phloem to the meristemic region (from 0−10*μ*m for a mature embryo [52] to about 80*μ*m for a 11 days old seedling [50]) suggests that advection via the phloem can dictate the length scale of the CK profile and hence the size of the meristemic region in a young plant.

#### Reproduction of the *clv3* mutant expression patterns

**Fig 6:**
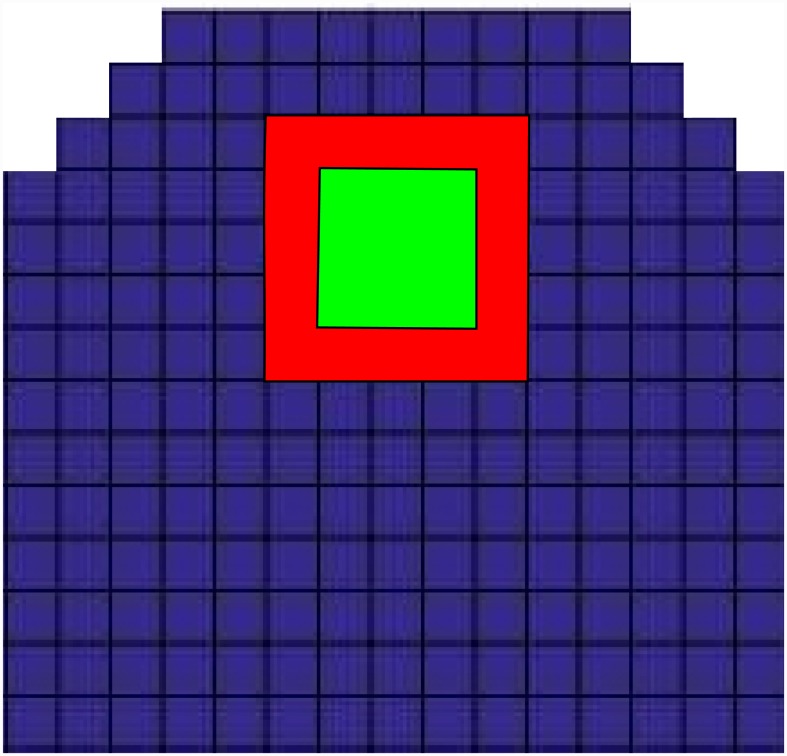
The effect of the *clv3* mutation on WUS expression. Green shows the extent of WUS expression in wildtype, and red shows WUS expression in the clv3 mutant. The expression zones are defined as cells that express WUS at the half maximum level of expression in the mutant or higher. In the mutant the concentration of WUS increases, this means the number of cells that express WUS at a high enough level to be considered within the expression zone, increases. For examples of models out put in an uncropped template see S2 Fig.

In the *clv3* mutant the WUS expression domain expands laterally. Additionally the concentration of WUS within its domain increases. The lateral expansion of WUS domain is accompanied by lateral expansion of the meristem as a whole [1]. Whether the WUS domain elongation happens as a result of meristem elongation or is the cause of it, or whether they are independent of each other, is not clear. In our simulations of the *clv3* mutant, the concentration of WUS increases within its domain and the expansion of the domain occurs in all directions, while the upward shifting of the WUS domain does not occur, Fig 6. This could result from the static nature of the model which does not consider cell division and/or elongation. In *clv3* mutant the meristem becomes enlarged in comparison to wildtype. For the vegetative meristem this enlargement appears to be around 20% [1] (compare Fig 5A and 5D in this reference). This does not affects the model output as the model is resistant to moderate changes in template size in both lateral and apical-basal direction, see S4 Fig.

#### Reproduction of the effect of laterally extending the meristem size

**Fig 7:**
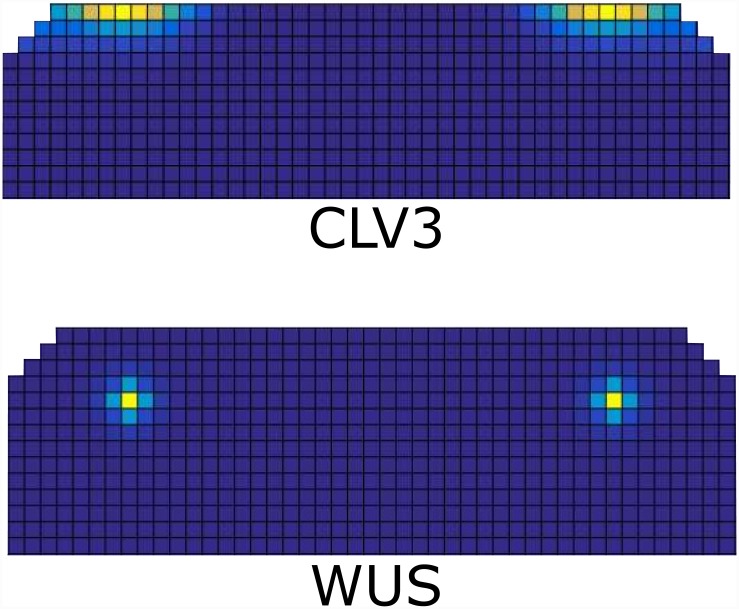
Expression of the molecules in the model, when meristem size is extended laterally. (A) two separate WUS expression centers form, (B) CLV3 expression zones form above each WUS center. For examples of models out put in an uncropped template see S2 Fig.

Graf *et al.* identified a mutant defective in shoot meristem development, called *mgol-4*. In mature *mgol-4* plants, the meristem is enlarged and becomes fragmented into multiple apices, each containing a separate stem cell niche. We simulated this effect by laterally extending the width of the meristem, as shown in Fig 7. *In vivo*, the mutant exhibits other developmental defects and the enlarged SAM does not possess a smooth and uniform edge, but forms a rather jagged and disorganized structure [53]. Our aim was to investigate wether the model, in general, is capable of generating multiple WUS centers in a larger domain. When the width of the domain is doubled, two WUS centers appear, the expression of WUS and CLV3 can be seen in Fig 7A and 7B. This multiplication of the pattern in a larger domain, is a known characteristic of reaction-diffusion systems [54], and further demonstrates the competence of a reaction-diffusion system in modeling the WUS regulation within the SAM.

#### The model exhibits regenerative ability of the meristem following laser ablation

**Fig 8:**
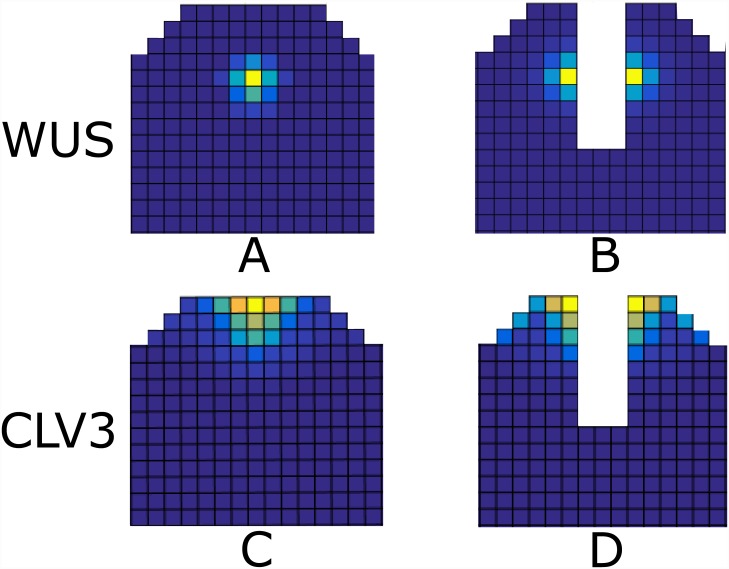
WUS and CLV3 expression patterns after *in silico* ablation. (A) and (C): the wildtype expression pattern of WUS and CLV3. (B) and (D): WUS and CLV3 expression patterns that form after ablation of the center of the SAM including the SCD and OC. For examples of models out put in an uncropped template see S2 Fig.

Experimental observations show that after the removal of the OC and stem cell domain (SCD) in the SAM of tomato via laser ablation, two new WUS centers form at the opposite sides of the ablation [55]. Starting from a wildtype expression pattern, Fig 8A and 8B), we eliminated the WUS and CLV3 expressing cells. When the system again reaches a steady state, two new OCs and SCDs form at the either side of the ablation site in a very similar manner to the experimental observation, (Fig 8C and 8D) as well as a previous model [26]. Such regenerative ability is an essential property of the SAM. The model predicts the presence of CK signaling and AHK4 at newly formed WUS expression centers after ablation. To our knowledge the presence of AHK4 expression patterns and CK activity have not been analyzed in the SAM after laser ablation. We therefore performed laser ablation experiments and tested for recovery on the level of CK signaling.

### Microsurgical and laser ablation experiments

**Fig 9:**
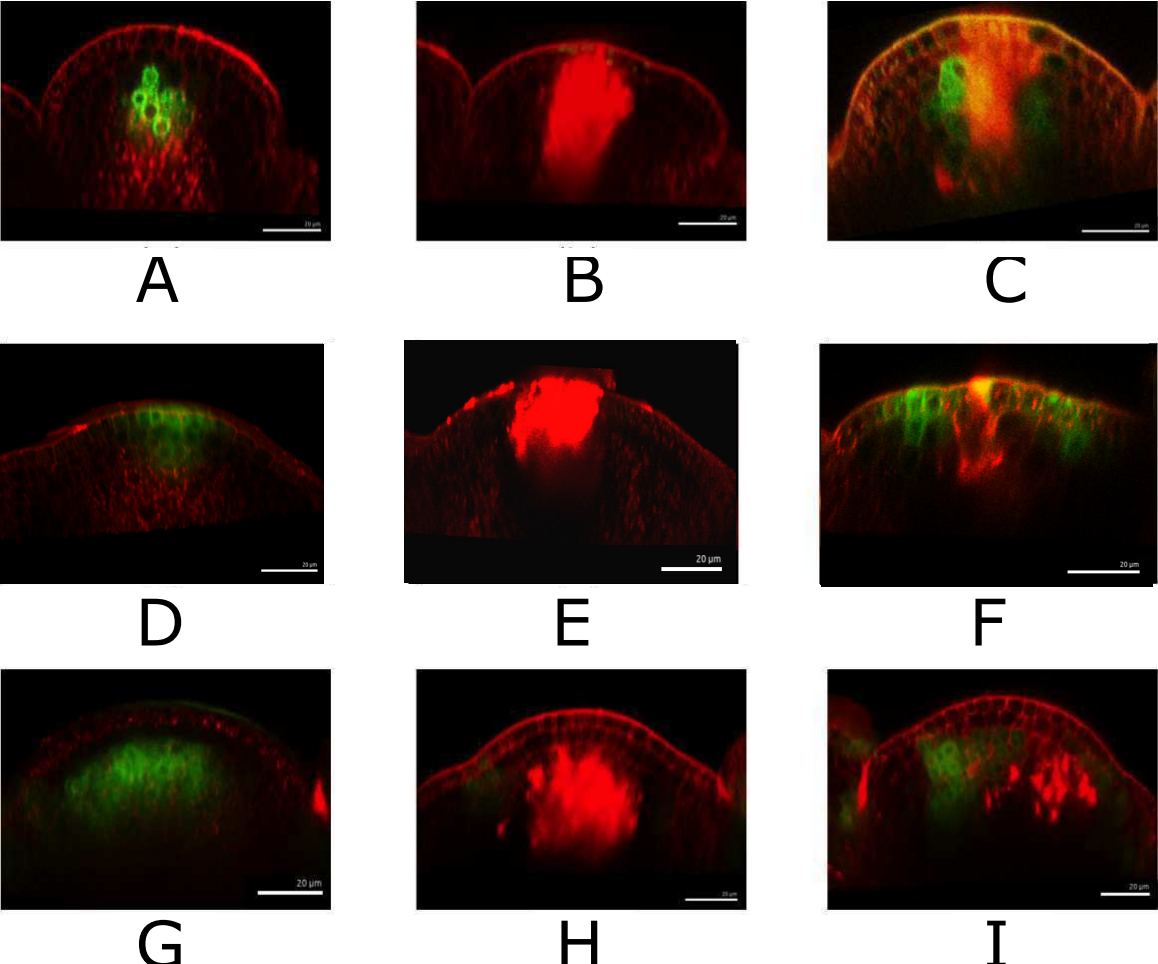
Laser ablation of *WUS*, *CLV3* and *TCS* domains. *WUS::GFP* promoter fusion expression before (A), just after (B) and 1 d after (C) laser ablation. *CLV3::GFP* expression before (D), just after (E) and 1 d after (F) laser ablation. *TCS* promoter expression before (G), just after (H) and 2 d (I) after laser ablation. The green signal is *WUS::GFP* in A, B, C, *CLV3::GFP* in D, E, F, *TCS* in G, H, I. Red signal is propidium-iodide (PI)-stained cell wall or laser ablated cells.

The model assumptions presented here suggest that the WUS expression in the OC is maintained via a reaction-diffusion network at the CK expression level. Laser ablation and microsurgical studies have shown that upon removing the WUS expressing cells the WUS expression is regenerated [55]. Our model further predicts that this recovery takes place at the level of CK signaling. In order to test this hypothesis, we performed microsurgical and laser ablation experiments, where we removed the cells within meristem that express the GFP under the *TCS* marker gene. We also performed the same experiment with GFP expressed under *WUS* promoter. In our initial trials, we observed that the expression of TCS fades away following the dissection of the meristem. This could be due to the lack of CK supply through the stem to the meristem. In order to compensate for the lack of supply of CK via the stem and to aid the visualization of the expression of TCS in the days following the dissection, we cultured the meristems after dissection in a CK containing medium. Our results demonstrate that the CK signaling domain within the meristem regenerates within 1–2 days following microsurgical ablation as shown in Fig 9G–9I, in a similar manner and time-frame as WUS expression, see Fig 9A–9C. Furthermore, we carried out ablation experiments on plants expressing *pclv3*-GFP. Upon removing the organizing center and the CLV3 expressing cells, it was observed that CLV3 becomes visible 3 days after the ablation, see Fig 9D–9F. The time-frame of recovery of CLV3 compared to WUS and TCS is in agreement with the model assumptions.

## Discussion

WUS is a major component of SAM development and stem cell homeostasis. Recent experimental evidence has revealed a diverse and extensive network comprising genes and hormones that contribute towards regulation of the SAM. Despite these findings, and several modeling efforts, it is still unclear how the WUS expression domain is restricted and centered within the SAM. We argue that the patterning and regulation of WUS within the SAM cannot be well understood without addressing the cell lineage-independent nature of it. The current knowledge, puts CK forward as a major factor in positioning and patterning of the SAM. We developed a model strictly based on the known mechanism of CK reception and signaling via the AHK4 receptor. Our experimental results show that, upon laser ablation of OC and SCD, the meristem as a whole is able to regenerate the WUS expressing cells as well as stem cells. In addition we demonstrate that the CK signaling domain within the SAM shows similar regenerative capabilities. Considering the time-frame of the recovery of WUS and TCS expression after ablation and the current understanding of the role of CK signaling in regulating the SAM activity, the experimental results suggest that the CK signaling could be the basis of the regenerative ability of the SAM as a whole. The time-frame of the regeneration of CLV3 compared to WUS and TCS, demonstrated that the recovery of CK signaling and subsequently the WUS expression is sufficient for the recovery of stem cell population.

Concepts such as L1 and factor X have been consistent features of activator-inhibitor-based modeling of SAM and their role in the work presented here is in principle same as earlier works. The main contribution of this work lies in the observation that some know components of CK signaling network have the capability of functioning as an activator-inhibitor system. By incorporating our assumptions of a diffusing inhibitor, a feedback loop involving the type-B ARRs and AHK4 and L1 signal, we demonstrate the potential of the CK signaling network in generating patterns within the SAM, in close agreement with the experimental observations. In addition our experimental results suggest that specification of OC including its self-organizing properties can arise, at least in part, via CK signaling. If type-A ARRs are assumed to be highly mobile, the intermediate factor X is not required and the model can function with type-A ARRs directly inhibiting the phosphorylation of type-B ARRs. We explicitly tested this scenario and observed, with adjustment of parameters the model is capable of producing the same patterns. In case the type-A ARRs do not fulfill the requirements, the proposed intermediate factor X is necessary. To our knowledge the studies focused on genetic regulation of the SAM do not put forward a candidate for factor X. While inhibiting type-B ARRs, factor X is predicted to have an overlapping maxima and expression domain with type-B ARRs. At first sight, AHP6, a well-known inhibitor of CK signaling [43] appears as a likely candidate. However its expression pattern does not match the predicted pattern for factor X [56, 57]. In the model factor X has a higher diffusion rate than other molecules, including the small CLV3 peptide. This hints at factor X being an even smaller molecule, perhaps a micro-RNA (miRNA). miRNA165/166, are mobile signaling molecules that suppress CK signaling via inhibition of CK production [58, 59]. However the expression pattern of these molecules is very different from the predicted expression of factor X [60]. It remains to be seen whether the new and active area of research on the role of miRNAs in plant genetic regulation would identify an experimental counterpart for this hypothetical molecule.

The inhibition of upstream CK signaling via type-A ARRs is essential for correct model output. This is because the aforementioned interaction constitutes a part of the core activator-inhibitor motif. In contrast the type-A ARR inhibiton of type-Bs via competition for phosphate is not necessary for correct model functionality, see S7 Fig. Our simulations show that the model can produce the corrects output in presence and absence of phosphate competition. While verification of the mechanism of type-A ARRs inhibitory effects are out of the scope of the model, the results suggest that the upstream inhibition is the main mechanism in meristem patterning.

One important ingredient of the model is the observation that the CK concentration profile is limited to the upper 20–25 cell layers. The exact cause of this is not important for the model to work, but as there is no evidence of a diffusion barrier for CK, we explored the consequences of a diffusion-like transport of CK within the tissue. Because the determining parameters for the CK profile are unknown, we cannot limit the synthesis regime of CK, besides the plausible assumption that CK synthesis is confined to the SAM. However, based on these considerations and using estimates for the effective diffusion rate of CK we conclude that the average lifetime of a CK molecules within the SAM is of the order of a few minutes. A further consequence of the model is that the size of the meristem might be determined by two distinct physical mechanisms. The model suggests that in adult plants the size of the SAM is governed by the length scale of CK diffusion, while in young plants it is determined by the distance from the L1 to the phloem.

We have shown that a combination of boundary driven patterns—CK and L1 signal acting as morphogenes—and a reaction diffusion system including AHK4 and its assumed inhibitor can account for a variety of observed phenomena regarding the SAM. The reorganization of the SAM after laser ablation and the appearance of multiple OCs upon lateral extension of the SAM closely resemble the properties of a reaction diffusion system. Our results suggest that both a short and a long range morphogene are required for establishment and regulation of WUS and CLV3 expression patterns within the SAM. The long ranged (on the scale of the SAM) CK confines the WUS expression peak to the SAM. The short ranged L1 signal is required in order to restrict the expression of CLV3. We show that L1 signal, originating from the L1 layer and diffusing downwards, can adequately explain the induction of CLV3 expression in a specific location at the tip of the SAM. The model predicts that the signal does not diffuse past the first few cell layers beneath the L1. The recently characterized miR394, produced at the L1 layer and necessary for establishment and regulation of stem cells by WUS, provides a suitable candidate for the role of L1 signal in the SAM. The proposed minimal model focuses on specific aspects in order to understand the concepts and is not expected to capture the complex biological system in its full detail. This is specifically true for redundancy, which is a common feature of many biological systems. A survey of literature reveals a high degree of redundancy within the CK sub-network. There are several types of CKs in plants. Many ARRs have similar expression patterns and are thought to be at least partially redundant [10]. Single and even multiple mutants deficient in CK biosynthesis do not show significant SAM phenotypes. The same is true for many type-A and type-B ARR genes. Therefore, single mutant phenotypes predicted by the reductionist model presented here, cannot be expected to correspond to the observed single mutant phenotypes. However, the model is expected to exhibit systemic behavior that could be used to assess the hypothesis under study. Furthermore, the model makes specific predictions that can be utilized to design experiments to test the model hypothesis and to further clarify underlying mechanism of gene expression patterning within the SAM. In summary, we show that regulation of the CK receptor AHK4, through a reaction-diffusion mechanism can plausibly account for an array of observed phenomena regarding WUS patterning and thus providing one possible answer to the question of how the organizing center is centered.

## Materials and Methods

### Plant material and shoot meristem culture

Plants and cultured apices were grown under the long photoperiod (16 h light). The following lines of *Arabidopsis* have been described previously: Arabidopsis TCS-GFP containing an enhanced version of the published construct [61] and WUS-GFP [62] are in the Col-0 background. CLV3-GFP [19], is in the Landsberg erecta (Ler) background. For in vitro *Arabidopsis* shoot meristem culture, inflorescence meristems of *Arabidopsis* plants were dissected and transferred to MS medium containing 0.7% phytagel. For CK treatment, benzyladenine (Sigma-Aldrich) was added to the medium at final concentration of 500 *μ*M.

In laser ablation experiments, a total of 6 WUS-GFP seedling were ablated and all subsequently recovered. Out of the 9 TCS-GFP seedling that were ablated and subsequently placed in BA medium 7 recovered. All of 5 CLV3-GFP seedling that were ablated subsequently recovered. In microsurgical experiments 2/2, 2/2 and 3/3 recovered for TCS-GFP, WUS-GFP and CLV3-GFP respectively.

### Microscopy and laser ablation

Confocal analysis was carried out using a Leica upright confocal laser-scanning microscope (Leica TCS SP5) with long-working distance water immersion objectives. The cell wall was stained with 0.2% propidium iodide (PI; Sigma-Aldrich) for 1min. Apices were observed in the 3% agarose medium. Following laser settings are used for the observation; GFP (Argon laser, excitation 488nm, emission, 500–530nm), PI-staining (Argon laser, excitation 488nm, emission 600–700nm). Poking was carried out by fine tungsten needles (World Precision Instruments). Laser ablation was carried out by diode laser at the wavelength of 405 nm. Target cells were chosen using the Leica bleach point function and submitted to UV laser irradiation (90% laser power for 25 seconds). Confocal z-stacks were 3D reconstructed by MorphographX software [63].

### Simulation details

Parameters were chosen from the biological and physical relevant ranges and adjusted to maximally approximate the available data. All simulation where carried out with arbitrary initial concentrations of all the molecules in the model, within the [0,0.5] range, unless otherwise stated. Simulations were continued until the steady state was reached.

### The diffusion operator

The diffusion operator $\hat{D}$ operating on the square grid index is defined by:

$$\begin{array}{ccc} \hat{D}C_{ij} & = & C_{i-1j}+C_{i+1j}+C_{ij+1}+C_{ij-1}-4C_{ij}. \end{array}$$

### L1 signal and CK profile

For both the L1 signal and CK the problem can be described as diffusion in one-dimensional semi-infinite space with finite production regime. We approach the problem by dividing the space into two section, a region where the signaling molecule is produced, and a region where the production of the signaling molecule does not occur.

For simplicity we treat space as continuous; the corresponding partial differential equation for the concentration *ϕ* of a diffusing molecule in steady state reads:

$$\begin{array}{ccc} D\frac{\partial^{2}\phi_{<}}{\partial x^{2}}-\lambda \phi_{<}+\Gamma & = & 0:\;x\leq x_{0}\\ D\frac{\partial^{2}\phi_{>}}{\partial x^{2}}-\lambda \phi_{>} & = & 0:\;x>x_{0}\\ D{\left.\frac{\partial \phi_{<}}{\partial x}\right|}_{x=0} & = & 0\\ \phi_{<}\left(x_{0}\right) & = & \phi_{>}\left(x_{0}\right)\\ D{\left.\frac{\partial \phi_{<}}{\partial x}\right|}_{x=x_{0}} & = & D{\left.\frac{\partial \phi_{>}}{\partial x}\right|}_{x=x_{0}}\\ \lim_{x\to \infty }\phi_{>}\left(x\right) & = & 0 \end{array}$$

In above equations *λ* is the degradation rate, Γ is the production rate of the signaling molecule in the production domain and *D* is the diffusion rate. Rescaling the spatial dimension with the typical length scale *L* for a cell in the SAM tissue, i.e. $\tilde{x}=x/L$, the solution to these equations are given by:

$$\begin{array}{ccc} \phi_{<}\left(\tilde{x}\right) & =\frac{\Gamma }{\lambda }\left(1-e^{-\frac{n_{0}}{\tilde{l}}}\cosh\left(\frac{\tilde{x}}{\tilde{l}}\right)\right) & \tilde{x}\leq n_{0} \end{array}$$ (9)

$$\begin{array}{ccc} \phi_{>}\left(\tilde{x}\right) & =\frac{\Gamma }{\lambda }e^{-\frac{\tilde{x}}{\tilde{l}}}\sinh\left(\frac{n_{0}}{\tilde{l}}\right) & \tilde{x}>n_{0} \end{array}$$ (10)

where $\tilde{l}:=\sqrt{d/(\lambda L^{2})}$ and *n*_0_ = *x*_0_/*L*. The L1/CK profile is given by *L1*_*ij*_ */CK*_*ij*_ = *ϕ*(*x* = *i*). The rescaled profile $\phi /\phi (\tilde{x}=0)$ is shown in S1 Fig for different values of *n*_0_ and $\tilde{l}$, corresponding to L1 and CK, resp.

We take the observation that CK profile is not observed after the 25th cell layer into account by requiring *CK*_*i* = 25*j*_/*CK*_*i* = 1*j*_ = 1/2. From this follows:

$$\begin{array}{ccc} 25-n_{0} & = & \tilde{l}\ln\left(1+\exp\left(-\frac{n_{0}}{\tilde{l}}\right)\right) \end{array}$$

which can only be solved numerically. Rewriting the root of this equation, $\tilde{l}=\tilde{l}\left(n_{0}\right)$, for the inverse degradation rate- *λ*^−1^ = *τ*, the average lifetime, finally yields the relation between the lifetime, synthesis range and diffusion constant:

$$\begin{array}{c} \tau \left(n_{0},d\right)=\frac{{\tilde{l}}^{2}\left(n_{0}\right)L^{2}}{d}. \end{array}$$

In Fig 5 we used *L* = 5*μm*, taken from [3].

### Role of advection

An interesting question in this context is, how the phloem might alter the CK profile. The phloem starts several cell layers below the meristem [50, 51]. A simple physical picture for the CK transport inside the phloem is that of mass transport due to the bulk motion of a fluid (advection). To study this problem we now divide the tissue into three zones: *x* ≤ *x*_0_: synthesis regime (synthesis + degradation + diffusion), *x*_*m*_ ≥ *x* > *x*_0_: diffusion regime (diffusion + degradation), and *x* > *x*_*m*_, advection regime (diffusion + advection + degradation). This is in steady state described by the following set of equations:

$$\begin{array}{ccc} D\frac{\partial^{2}\phi_{1}}{\partial x^{2}}-\lambda \phi_{1}+\Gamma & = & 0:\;x\leq x_{0}\\ D\frac{\partial^{2}\phi_{2}}{\partial x^{2}}-\lambda \phi_{2} & = & 0:\;x_{m}\geq x>x_{0}\\ D\frac{\partial^{2}\phi_{3}}{\partial x^{2}}-v\frac{\partial \phi_{3}}{\partial x}-\lambda \phi_{3} & = & 0:\;x>x_{m}\\ D{\left.\frac{\partial \phi_{1}}{\partial x}\right|}_{x=x_{0}} & = & D{\left.\frac{\partial \phi_{2}}{\partial x}\right|}_{x=x_{0}}\\ D{\left.\frac{\partial \phi_{2}}{\partial x}\right|}_{x=x_{m}} & = & D{\left.\frac{\partial \phi_{3}}{\partial x}\right|}_{x=x_{m}}-v\phi_{3}\left(x_{m}\right) \end{array}$$

In addition we require that the system is closed at *x* = 0 and that the solution is continuous and vanishes at infinity:

$$\begin{array}{c} {\left.\frac{\partial \phi_{1}}{\partial x}\right|}_{x=0}=0;\;\phi_{1}\left(x_{0}\right)=\phi_{2}\left(x_{0}\right);\;\phi_{2}\left(x_{m}\right)=\phi_{3}\left(x_{m}\right);\;\lim_{x\to \infty }\phi_{3}\left(x\right)=0. \end{array}$$

In order to further analyze this, we rescale length again with the typical cell size *L*. Also, we rescale the concentrations *ϕ*_*i*_ with *ϕ*_0_ = Γ/*λ* and finally arrive at:

$$\begin{array}{ccc} l^{2}\frac{\partial^{2}\phi_{1}}{\partial {\tilde{x}}^{2}}-\phi_{1}+1 & = & 0:\;\tilde{x}\leq n_{0}\\ l^{2}\frac{\partial^{2}\phi_{2}}{\partial {\tilde{x}}^{2}}-\phi_{2} & = & 0:\;{\tilde{x}}_{m}\geq \tilde{x}>n_{0}\\ l^{2}\frac{\partial^{2}\phi_{3}}{\partial {\tilde{x}}^{2}}-l_{a}\frac{\partial \phi_{3}}{\partial \tilde{x}}-\phi_{3} & = & 0:\;\tilde{x}>{\tilde{x}}_{m}\\ {\left.\frac{\partial \phi_{1}}{\partial \tilde{x}}\right|}_{\tilde{x}=n_{0}} & = & {\left.\frac{\partial \phi_{2}}{\partial \tilde{x}}\right|}_{\tilde{x}=n_{0}}\\ l^{2}{\left.\frac{\partial \phi_{2}}{\partial \tilde{x}}\right|}_{\tilde{x}={\tilde{x}}_{m}} & = & l^{2}{\left.\frac{\partial \phi_{3}}{\partial \tilde{x}}\right|}_{\tilde{x}={\tilde{x}}_{m}}-l_{a}\phi_{3}\left({\tilde{x}}_{m}\right) \end{array}$$

with *l*_*a*_ = *v*/(*Lλ*). For large Péclet numbers *l*_*a*_/*l*^2^ = *vL*/*D* ≫ 1 the flux continuity equation at $\tilde{x}={\tilde{x}}_{m}$ simplifies to $\phi_{3}\left({\tilde{x}}_{m}\right)=0$ and by using the continuity of the solution we find: $\phi_{2}\left({\tilde{x}}_{m}\right)=0$, which yields a closed set of equations for $\tilde{x}\leq {\tilde{x}}_{m}$. For a phloem flux of *v* = 50*μ*m/s [64] the Péclet number would be roughly in the range 6 > *l*_*a*_/*l*^2^ > 1. Using the approximation for large Péclet numbers, we find:

$$\begin{array}{ccc} \phi_{1}\left(\tilde{x}\right) & =1-\cosh\left(\frac{\tilde{x}}{l}\right)\frac{\cosh\left(\frac{{\tilde{x}}_{m}-n_{0}}{l}\right)}{\cosh\left(\frac{{\tilde{x}}_{m}}{l}\right)} & \tilde{x}\leq n_{0}\\ \phi_{2}\left(\tilde{x}\right) & =\sinh\left(\frac{{\tilde{x}}_{m}-\tilde{x}}{l}\right)\frac{\sinh\left(\frac{n_{0}}{l}\right)}{\cosh\left(\frac{{\tilde{x}}_{m}}{l}\right)} & {\tilde{x}}_{m}\geq \tilde{x}>n_{0}\\ \phi_{3}\left(\tilde{x}\right) & =0 & \tilde{x}>{\tilde{x}}_{m}\; \end{array}$$

In adult plants the closest distance to the phloem was measured to be roughly 220*μ*m [50, 51], which translates to ${\tilde{x}}_{m}\approx 44$. Corrections to the estimate of the lifetime of CK are of the order $O(e^{-2{\tilde{x}}_{m}/l_{0}})$, where *l*_0_ is the root of the equation *CK*_*i* = 25*j*_/*CK*_*i* = 1*j*_ = 1/2 without advection, i.e., *l*_*a*_ = 0. Using ${\tilde{x}}_{m}\approx 44$ and *l*_0_ ≈ 32 we find $e^{-2{\tilde{x}}_{m}/l_{0}}\approx 0.06$. We conclude that the phloem has no significant impact on our estimates for the lifetime of CK.

### Activation of the ARRs

The AHK4 receptor exhibits the interesting feature that it works as a kinase as well as a phosphatase depending on whether CK is bound to it or not [65]. The other interesting feature is that phosphorylation of the type-A and type-B ARRs is not directly but through phosphotransfer proteins, the AHP family. For simplicity we assume in the following derivation of the transfer function that the CK signaling, i.e., binding of CK to AHK4, binding of AHP to AHK4, phosphorylation of AHP, phosphorylation of the ARRs are sufficiently fast processes compared to the time scale of gene expression. Moreover, we consider the extracellular binding of CK to AHK4 as being independent of the intracellular binding of AHP. Using the quasi-steady state assumption and suppressing for notational simplicity the spatial index on the concentrations we find for the amount of receptors occupied by CK: *R*_*b*_ = *αCKR*(1+*αCK*)^−1^ and for the unoccupied receptors: *R*_*f*_ = *R*(1+*αCK*)^−1^, where *CK* denotes the CK concentration (we neglect the depletion of the free CK by binding to AHK4), *R* is the concentration of AHK4, and *α* is the corresponding inverse *K*_*d*_ value of the binding reaction. Assuming further a surplus of AHP phosphotransfer proteins compared to the amount amount of AHK4 receptors, the abundance *H*_*p*_ of phosphorylated AHPs is given by: *H*_*p*_ = *γR*_*b*_(1+*βR*_*f*_)^−1^, where *γ* and *β* describe the kinase and phosphatase activity of the receptor, respectively. The phosphorylated AHP (*H*_*p*_) can bind either to type-A (*A*) or type-B ARR (*B*), hence *A* and *B* compete with each other for *H*_*p*_. Using again the quasi steady-state assumption we find for the fraction of phosphorylated *A*:

$$\begin{array}{c} \frac{A_{p}}{A}=\sigma_{A}\frac{H_{p}}{1+\sigma_{A}A+\sigma_{B}B}. \end{array}$$

Here *σ*_*A*_ and *σ*_*B*_ are the inverse *K*_*d*_ values for the binding reactions for *A* and *B*, respectively. Inserting the above results for *H*_*p*_, *R*_*b*_, and *R*_*f*_ we arrive at:

$$\begin{array}{c} \frac{A_{p}}{A}=\sigma_{A}\alpha \gamma \frac{\text{Ck}R}{\left(1+\alpha \text{Ck}+\beta R\right)\left(1+\sigma_{A}A+\sigma_{B}B\right)}. \end{array}$$

To obtain non-dimensional quantities we rescale all concentrations with the half-maximal concentration $W_{\frac{1}{2}}$ of the CLV3 activation by WUS, Eq (6). Defining: $k_{2}:=\alpha W_{\frac{1}{2}}$, $k_{3}:=\beta W_{\frac{1}{2}}$, $k_{4}:=\sigma_{A}W_{\frac{1}{2}}$, $k_{5}:=\sigma_{B}W_{\frac{1}{2}}$ yields:

$$\begin{array}{c} \frac{A_{p}}{A}∼\Gamma . \end{array}$$

where Γ is the transfer function for describing the two-step phosphorelay given in Eq (8). Following the suggestion in the literature [65] that the phosphotransfer to the type-B ARR is inhibited by phosporylated type-A ARR, we assume that a non cell-autonomous inhibitor *X*, which is activated by *A*_*p*_, inhibits the phosphorelay to type-B ARR non-competitively:

$$\begin{array}{c} \frac{B_{p}}{B}=∼\frac{\Gamma }{1+k_{6}X}. \end{array}$$

Rescaling time with the inverse degradation rate of WUS, the other terms in Eqs (1)–(7) follow directly from the assumptions described in Results section.

### Model evaluation

In order to evaluate the resulting simulation patterns one needs to define appropriate quantitative measures. In order to evaluate the output of the model, quantitative measures must be defined based on experimental observations of WUS and CLV3 pattern in the SAM. In evaluating the model output we are concerned with general patterning capabilities rather than reproduction of experimentally observed patterns in detail. Therefore we focus on essential features that define the existence of the correct pattern. This allows for variability in model output by not imposing a too strict of a criteria for the correct pattern. The output of the model can be assessed using the WUS and CLV3 concentration distributions. We define the following marginalized distributions:

$$\begin{array}{ccc} {\bar{W}}_{i}\left(\vec{p}\right) & = & \frac{\sum_{j\in \Omega }W_{ij}\left(\vec{p}\right)}{∥\sum_{j\in \Omega }W_{ij}\left(\vec{p}\right)∥} \end{array}$$ (11)

$$\begin{array}{ccc} {\bar{C}}_{i}\left(\vec{p}\right) & = & \frac{\sum_{j\in \Omega }C_{ij}\left(\vec{p}\right)}{∥\sum_{j\in \Omega }C_{ij}\left(\vec{p}\right)∥} \end{array}$$ (12)

where ‖.‖ denotes the L2 norm. The cell indices *i* and *j* are restricted to the integration domain *Ω* which consists of the uppermost 9 cell layers and the central region of the meristem; *Ω* = {(*i*,*j*)|3 < *i* ≤ 12;2 < *j* ≤ 11}. These profiles adequately capture the distinguishing features of the patterns and can be obtained from the experimental data in the same manner (S5A Fig).

### Cost function construction

A set of reference marginalized and normalized concentrations of WUS and CLV3 can be obtained from experimental data as described in [66]; GFP intensity is used as a proxy for the concentration of WUS and CLV3 in an apical-basal cross-section of the 3D confocal stacks of SAM in pWUS-n3GFP and pCLV3-n3GFP respectively.

Using the two marginalized distributions given in Eqs (11) and (12), we define two objectives as the distances between the marginalized profiles:

$$\begin{array}{c} d_{W}\left(\vec{p}\right)=∥\bar{W}\left(\vec{p}\right)-{\bar{W}}_{\text{ref}}∥\\ d_{C}\left(\vec{p}\right)=∥\bar{C}\left(\vec{p}\right)-{\bar{C}}_{\text{ref}}∥. \end{array}$$

${\bar{W}}_{\text{ref}}$ and ${\bar{C}}_{\text{ref}}$ are the experimentally obtained marginalized reference concentration profiles. We aggregate the two objectives into a single cost function:

$$\begin{array}{c} L\left(\vec{p}\right)=d_{W}\left(\vec{p}\right)+d_{C}\left(\vec{p}\right). \end{array}$$

### Model parameters

S1 Table lists the non-dimensionalized parameters used in simulations presented in the main text unless otherwise stated.

### Parameter survey

Focusing on parameter set, ${\vec{p}}_{1}$, described in S1 Table, we define a hypercube *Ω* on the logarithmic scale, where we extended each parameter one order of magnitude in each direction: $\Omega =\Pi_{j}\left[p_{1}^{j}/10,10p_{1}^{j}\right]$. Within this hypercube a total of *N* = 2.5×10^5^ parameter sets were generated. For each parameter set $\vec{p}\in \left\{{\vec{p}}_{1},{\vec{p}}_{2},...,{\vec{p}}_{N}\right\}$ we calculated a score $L(\vec{p})$, as described above. The subset defined by $\omega =\{\vec{p}\in \left\{{\vec{p}}_{1},{\vec{p}}_{2},...,{\vec{p}}_{N}\right\}|L\left(\vec{p}\right)<0.1\}$ was obtained. Subset *ω* consists of parameter sets whose outputs are consistent with experimental observations. The threshold of 0.1 allows for variation in model output, while it insures the existence of the correct patterns.

### Sensitivity analysis

To characterize the effect of perturbations on model output a local sensitivity analysis was carried out. The normalized sensitivity of parameter $p_{i}^{j}$ belonging to the parameter point ${\vec{p}}_{i}$ is defined by:

$$\begin{array}{c} S_{i}^{j}=\frac{p_{i}^{j}}{L({\vec{p}}_{i})}\left|\frac{L\left(p_{i}^{1},\cdots ,p_{i}^{j-1},p_{i}^{j}+\delta ,p_{i}^{j+1},\cdots \right)-L\left({\vec{p}}_{i}\right)}{\delta }\right|. \end{array}$$

From the set of $S_{i}^{j}$ we calculated the quartiles as shown in the box-plot S6 Fig. [For the sensitivity analysis *δ* = 0.01 was used.

## Supporting Information

S1 FigBoundary layer profiles.Re-scaled (*ϕ*/*ϕ*_0_, *ϕ*_0_ = *ϕ*(0)) concentration profiles as given by Eqs (9) and (10) of the L1 (red) signal and CK (blue). The distance from the L1 layer is measured in units of the average cell size within the meristem. Parameters used: *n*_0_ = 1, $\tilde{l}=4$ for L1 and *n*_0_ = 8, $\tilde{l}=30$ for CK concentration profiles.(EPS)

S2 Fig Examples of model output on the full simulation grid. The full simulation template constitutes a 45x60 rectangular grid. The concentration of the molecules approaches zero at the template boundary.
(EPS)

S3 Fig WUS mobility is required for correct patterning of WUS. (A) Model simulation of wildtype WUS pattern. (B) Model simulation of WUS pattern when WUS mobility is set to zero in the model. (C) Wildtype WUS, CLV3 and L1 signal profiles along the center-line of the meristem (as shown in (A) and (B)). WUS domain extends to L1 layer. CLV3 pattern has a maximum at L1 layer. (D) Effects of WUS immobility in the model; WUS is not present in the upper cell layers of the SAM and CLV3 domain is severely reduced and misplaced.
(EPS)

S4 Fig Simulation output in alternative templates and L1 layer assignment. WUS and CLV3 patterns resulting from model simulations in alternative templates. are qualitatively unchanged compared to wildtype. (A) The original template coupled with an alternative implementation of L1 layer. (B) Extension of meristem by one cell layer in apical direction.(C) A simple rectangular implementation of the meristemic dome.
(EPS)

S5 Fig Scoring function objectives. (A) The scoring objectives focus on the a region of the cellular grid that corresponds to the SCD and OC. From this a marginalized WUS/CLV3 concentration profile is obtained. (B) A reference WUS/CLV3 concentration is obtained from the experimental data. (C) The comparison of these two profiles provides a measure of the model output against the experimentally observed patterns of WUS/CLV3 in the SAM.
(EPS)

S6 Fig Parameter sensitivity. The plot displays the sensitivity of model parameters to small perturbations. Most parameter show little sensitivity, while *k*_1_,*k*_6_,*k*_14_ and *d*_2_ show highest levels of sensitivity. In each box the central red line is the median. Edges of the box are the 25th and 75th percentiles. The whiskers show the range for data points that are not considered outliers. The red dots outside of this range are individual outliers. The parameter sets generated by the parameter survey and the sensitivity analysis, are all within the Turing space.
(EPS)

S7 Fig Phosphate competition between type-B and type-A ARRs does not significantly affect the model output. The difference in Model simulation of WUS and CLV3 expression, when phosphate competition between ARRs is absent from the model. The expression profile of WUS and CLV3 in absence of phosphate competition were subtracted from the corresponding wildtype expression profile. The expression profiles were normalizes to maximum levels in each case and the absolute difference between the two was calculated. To simulate the absence of phosphate competition, parameters *k*_4_ and *k*_5_ are set to zero. Model output is not significantly changed in absence of the phosphate competition.
(EPS)

S1 Table The Parameters used in the main text simulations. All parameters are dimensionless.
(TIFF)
