# Correction: Centering the Organizing Center in the *Arabidopsis thaliana* Shoot Apical Meristem by a Combination of Cytokinin Signaling and Self-Organization

**DOI:** 10.1371/journal.pone.0154638

**Published:** 2016-04-26

**Authors:** The PLOS ONE Staff

There is an error in Fig 1. The publisher apologizes for the error. Please see the corrected Fig 1 here.

**Fig 1:**
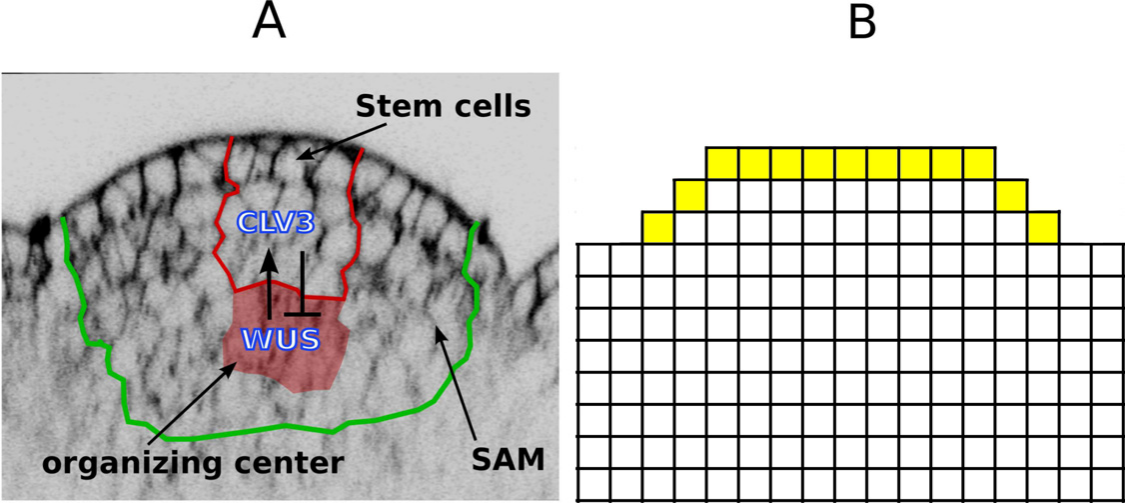
SAM architecture and its representation in the model. (A) An image of the SAM and the immediate surrounding area. The regions of interest are marked with colored boundaries. (B) Schematic representation of WUS and CLV domains; the three dimensional SAM, consisting of cells of various shapes and sizes, is modeled by a two dimensional grid consisting of identical blocks representing cells. The field of cells extend farther in lateral and basal directions. S2 Fig depicts the complete cellular template used for simulations.

## References

[1] Adibi M, Yoshida S, Weijers D, Fleck C (2016) Centering the Organizing Center in the *Arabidopsis thaliana* Shoot Apical Meristem by a Combination of Cytokinin Signaling and Self-Organization. PLoS ONE 11(2): e0147830 doi:10.1371/journal.pone.0147830 26872130 10.1371/journal.pone.0147830 PMC4752473

