# Correction: Centering the Organizing Center in the *Arabidopsis thaliana* Shoot Apical Meristem by a Combination of Cytokinin Signaling and Self-Organization

**DOI:** 10.1371/journal.pone.0174751

**Published:** 2017-03-23

**Authors:** Milad Adibi, Saiko Yoshida, Dolf Weijers, Christian Fleck

The affiliation for the first author is missing. Milad Adibi is affiliated with: LifeGlimmer GmbH, Markelstrasse 38, 12163 Berlin, Germany.

The following information is missing from the Funding section: LifeGlimmer GmbH provided funding in the form of salary to MA. The specific role of this author is articulated in the 'author contributions' section. The funder had no role in study design, data collection and analysis, decision to publish, or preparation of the manuscript.

The following information is missing from the Competing Interests section: MA is affiliated with LifeGlimmer GmbH. This does not alter our adherence to PLOS ONE policies on sharing data and materials.
