# Supplementary figures and images for: Centering the Organizing Center in the Arabidopsis thaliana Shoot Apical Meristem by a Combination of Cytokinin Signaling and Self-Organization
Source: PLoS One. 2016 Feb 12;11(2):e0147830. doi: 10.1371/journal.pone.0147830 (PMC4752473; doi:10.1371/journal.pone.0147830)

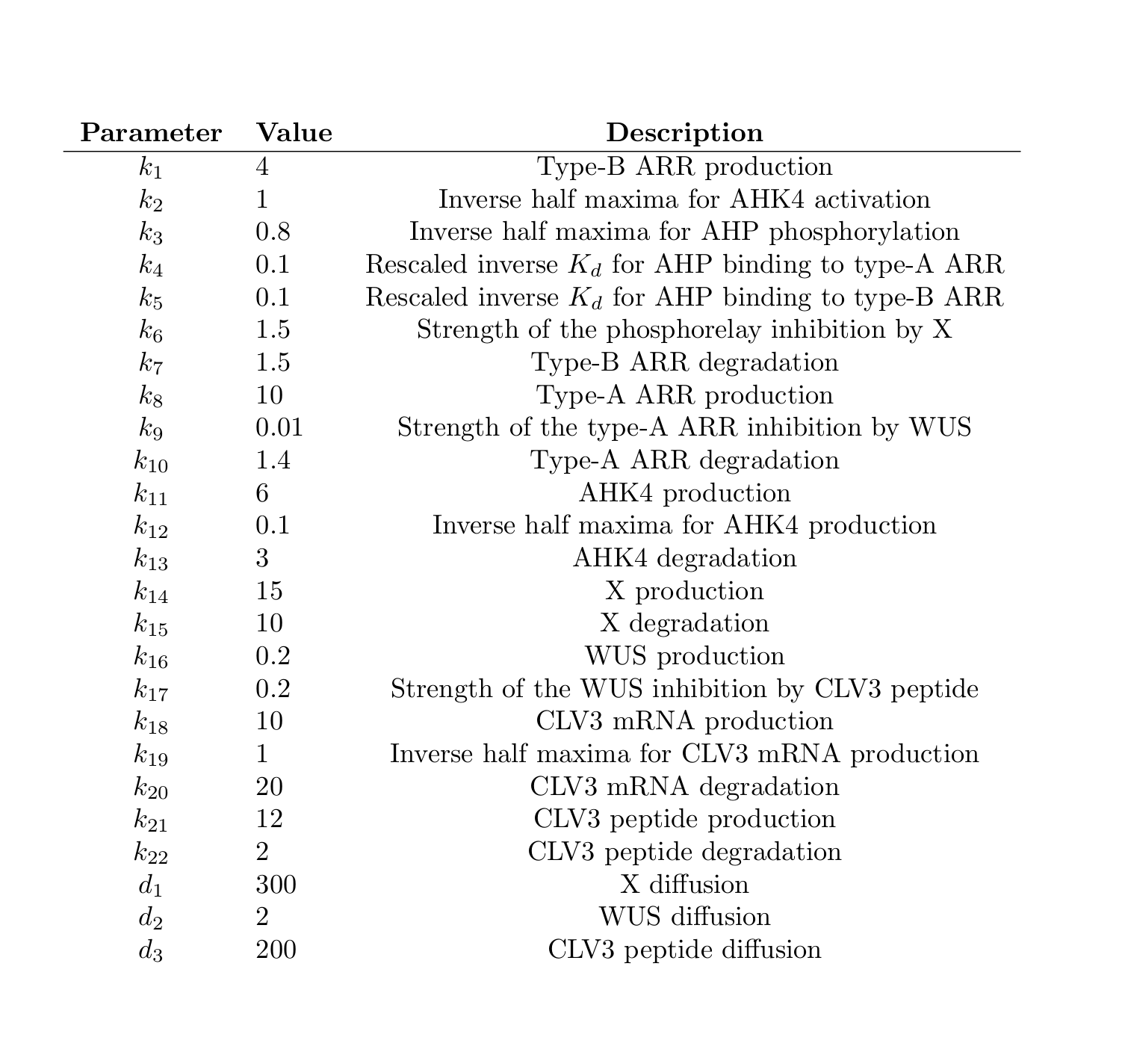

Supplement: S1 Table — All parameters are dimensionless. (TIFF) [file pone.0147830.s008.tiff]
